# *Cherax snowden*, a new species of crayfish (Crustacea, Decapoda, Parastacidae) from the Kepala Burung (Vogelkop) Peninsula in Irian Jaya (West Papua), Indonesia

**DOI:** 10.3897/zookeys.518.6127

**Published:** 2015-08-24

**Authors:** Christian Lukhaup, Jörn Panteleit, Anne Schrimpf

**Affiliations:** 1 Waldstrasse 5a, 66999 Hinterweidenthal, Germany; 2 Conservation Genetics,Institute for Environmental Sciences,University Koblenz-Landau, Fortstrasse 7, 76829 Landau, Germany

**Keywords:** Crustacea, Decapoda, Parastacidae, *Cherax snowden* new species, freshwater crayfish, Oinsok River Drainage, Kepala Burung (Vogelkop) Peninsula, Irian Jaya, Indonesia, West Papua, pet trade

## Abstract

A new species, *Cherax snowden* **sp. n.**, from the Oinsok River Drainage, Sawiat District in the central part of the Kepala Burung (Vogelkop) Peninsula, West Papua, Indonesia, is described, figured and compared with the closest related species, *Cherax holthuisi* Lukhaup & Pekny, 2006. This species is collected and exported for ornamental purposes and its commercial name in the pet trade is “orange tip” or “green orange tip”. Both species may be easily distinguished morphologically or by using sequence divergence, which is substantial, for considering *Cherax snowden* **sp. n.** to be a new species.

## Introduction

**Figure 1.**
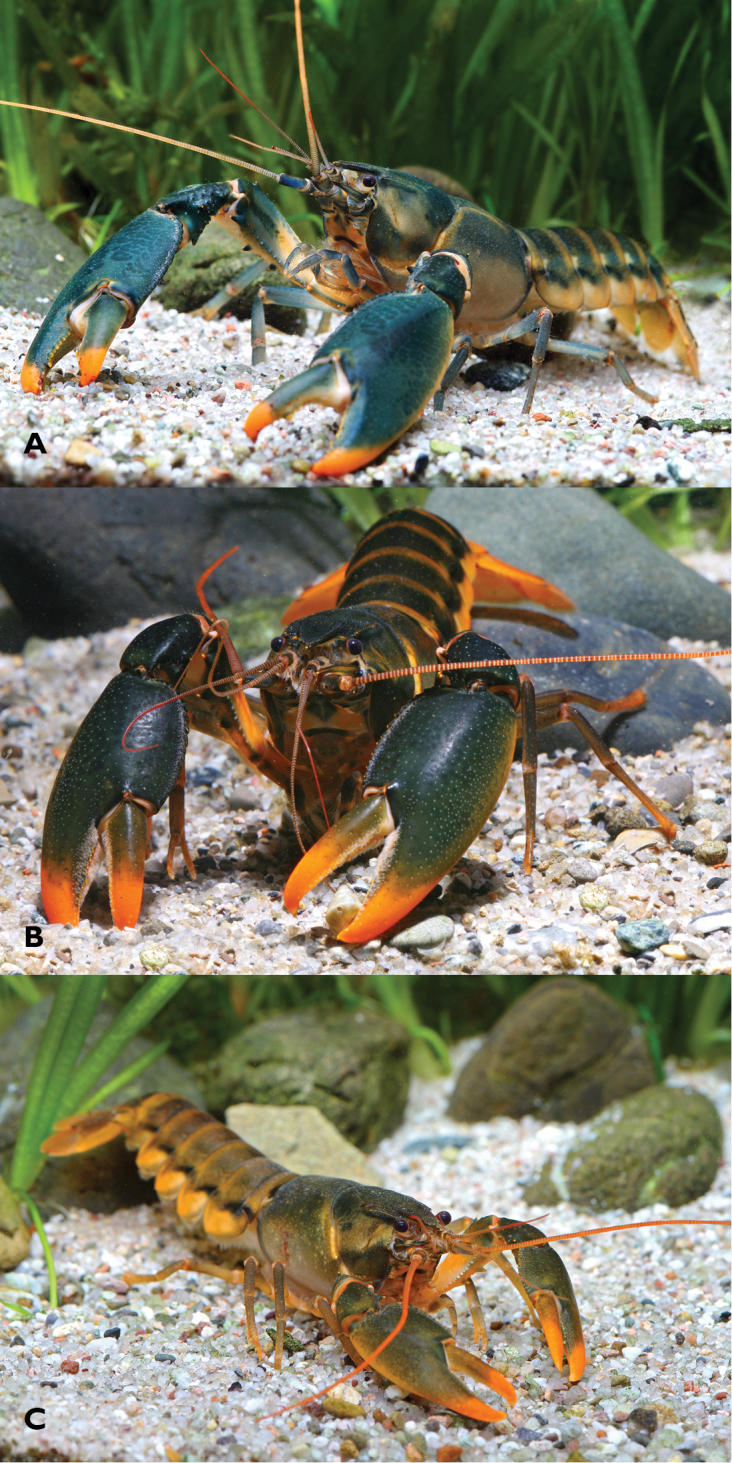
*Cherax snowden* sp. n. **A** paratype male (MZB Cru 4292) from Oinsok River drainage, Sawiat District **B** male from aquarium import (not listed in material examined) from Indonesia **C** Paratype female (MZB Cru 4293) from a unnamed tributary of the Oinsok River drainage.

The crayfish of the island of New Guinea were extensively studied by Holthuis (1949, 1956, 1958, 1982, 1986, 1996), with additions by Lukhaup and Pekny (2006, 2008a), Lukhaup and Herbert (2008), Lukhaup (2015), and Patoka et al. (2015). Nevertheless, over the last decade, there has been an increasing number of colourful crayfish, presumed to be a further undescribed species, sold from New Guinea in the ornamental fish trade in Europe and Asia under the names *Cherax* “orange tip”, and “green orange tip” (Lukhaup and Pekny 2008b, 2014). These have been exported to some countries in Europe, East Asia and America. Among the most common and popular colour forms are: (1) green, orange and yellow morph with orange tips (Fig. 1A–B); and (2) a greenish orange morph (Fig. 1C). While they are clearly species of *Cherax*, a large genus of freshwater crayfish occurring in Indonesia (West Papua), Papua New Guinea and Australia, their exact provenances could not be ascertained, with dealers claiming they came from Ajamaru (West Papua) and other places in the area that could not be confirmed. In the present contribution, this species is described as new to science and it is established that it is in fact native to the Oinsok River Drainage, Sawiat District of the Kepala Burung (Vogelkop) Peninsula, West Papua, Indonesia. The new species, *Cherax snowden*, differs from all other crayfish of this genus in the shape of its rostrum, shape of body and chelae and also in its colouration. *Cherax snowden* sp. n. is genetically and morphologically most similar to *Cherax holthuisi*, collected from the Kais River Drainage and Aitinjo Lake, Irian Jaya, Indonesia (Lukhaup and Pekny 2006).

These species may easily be distinguished on the basis of sequence divergence or by their colour and colouration pattern.

## Material and methods

The first specimens of the new species where exported from the city of Sorong, Indonesia as *Cherax* sp. in 2006. Those crayfish have been captured by a unknown local collector from Kepala Burung for ornamental purposes in West Papua, Indonesia and imported to Aquarium Dietzenbach /Germany through Maju Aquarium / Jakarta. Several animals from the first import were photographed and then preserved in 70% ethanol. Due to their colouration the first author named them *Cherax* sp. “orange tip” and “green orange tip”. In April 2015 we received another 6 animals through Garnelio, a leading german online store specialized in freshwater invertebrates from Mannheim, Germany. Furthermore, we recieved 20 additional specimens from Aquazone Indonesia a wholesalor for freshwater fish and freshwater invertebrates through Garnelio. The name of the crayfish collector in Sawiat District collecting for Aquazone Indonesia and other wholesaler in Indonesia is Irianto Wahid. According to the information obtained from Maju Aquarium and Aquazone Indonesia as well as from Irianto Wahid all specimens originated from creeks in the Sungai River Drainage. Two of the six animals obtained from Garnelio where photographed.All of them have been kept alive separately in aquarium tanks until samples of haemolymph were obtained for DNA analysis. After this procedure, the specimens were compared to the animals imported in 2006. They matched perfectly. They were subsequently preserved in 80% ethanol. One male from the shippment of April 2015 was selected as holotype, one female from the same shippment as allotype, another male as paratype.

DNA was extracted from muscle tissue using a standardized protocol (‘High Salt DNA Extraction Protocol for removable samples’; Alijanabi and Martinez 1997). A 600 base pair (bp) long fragment of cytochrome c oxidase subunit I (COI) of mitochondrial DNA, was ampliﬁed using the primer pair LCO1490 (5’-ggtcaacaaatcataaagatattgg-3’) and HCO2198 (5’-taaacttcagggtgaccaaaaaatca-3’) (Folmer et al. 1994). The polymerase chain reactions (PCR) were performed in a total volume of 20 µl, containing 0.125 µl GoTaq DNA Polymerase (Promega, Mannheim, Germany), 4 µl 5x Colorless GoTaq Flexi Buffer (Promega, Mannheim, Germany), 1.2 µl of 25 mM MgCl_2_ (Promega, Mannheim, Germany), 0.4 µl of 25 mM/l dNTPs (Fermentas, St. Leon-Rot, Germany), 0.8 µl of both primers with a concentration of 10 pmol/µl and 2 µl of the sample DNA. The following PCR-program was used: 4 min at 94 °C followed by 30 cycles each with 45 s at 94 °C, 45 s at 47 °C and 1 min at 72 °C. The final extension time was 10 min at 72 °C. Afterwards, the PCR-products were stored at 8 °C. PCR products were sequenced on a 3730 DNA Analyzer eight capillary sequencer (Applied Biosystems, MA, USA) by the company SeqIT (Kaiserslautern, Germany). Sequences were edited with Geneious 7.1.7 software (Biomatters Ltd.). The sequence will be submitted to GenBank after acceptance of the manuscript.

Additional we have downloaded the following sequences from GenBank: HG942364 – Cherax (Astaconephrops) quadricarinatus (von Martens, 1868), KJ950502 – *Cherax bicarinatus* (Gray, 1845), KJ950510 – *Cherax communis* Holthuis, 1949, KJ950520 – *Cherax holthuisi*, KJ950526 – *Cherax murido* Holthuis, 1949, KJ950529 – *Cherax paniaicus* Holthuis, 1949, KJ950533 – *Cherax peknyi* Lukhaup & Herbert, 2008, KJ950507 – Cherax (Astaconephrops) boesemani Lukhaup & Pekny, 2008, KM501043 – *Cherax* sp. and as an outgroup we used NC_026214.1 – *Euastacus spinifer* (Heller, 1865) and HG799087 – *Cherax destructor* Clark, 1936. All sequences were aligned with Geneious. We used jModelTest (Darriba et al. 2012) to estimate the best nucleotide substitution model and the HKY+G model was selected by Bayesian information criterion (BIC). Phylogenetic relationships were reconstructed using MrBayes 3.2.1 (Ronquist and Huelsenbeck 2003) as implemented in Geneious. We ran four independent chains of 10 million generations with a subsample frequency of one thousand after a burn-in period of 1 million.

## Systematics

### Family Parastacidae Huxley, 1879 Genus *Cherax* Erichson, 1846

#### Cherax snowden sp. n.

Taxon classificationAnimaliaDecapodaParastacidae

http://zoobank.org/136302EA-EEEA-4815-A5C3-6E37F29442A0

**Figure 2.**
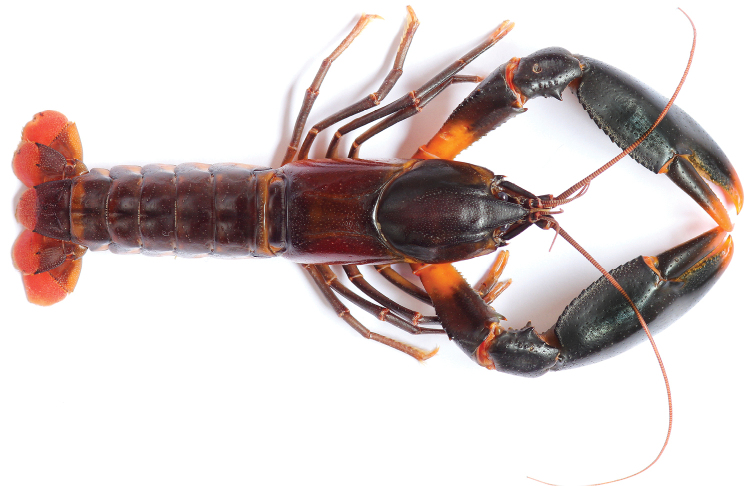
*Cherax snowden* sp. n. holotype male (MZB Cru 4291).

**Figure 3.**
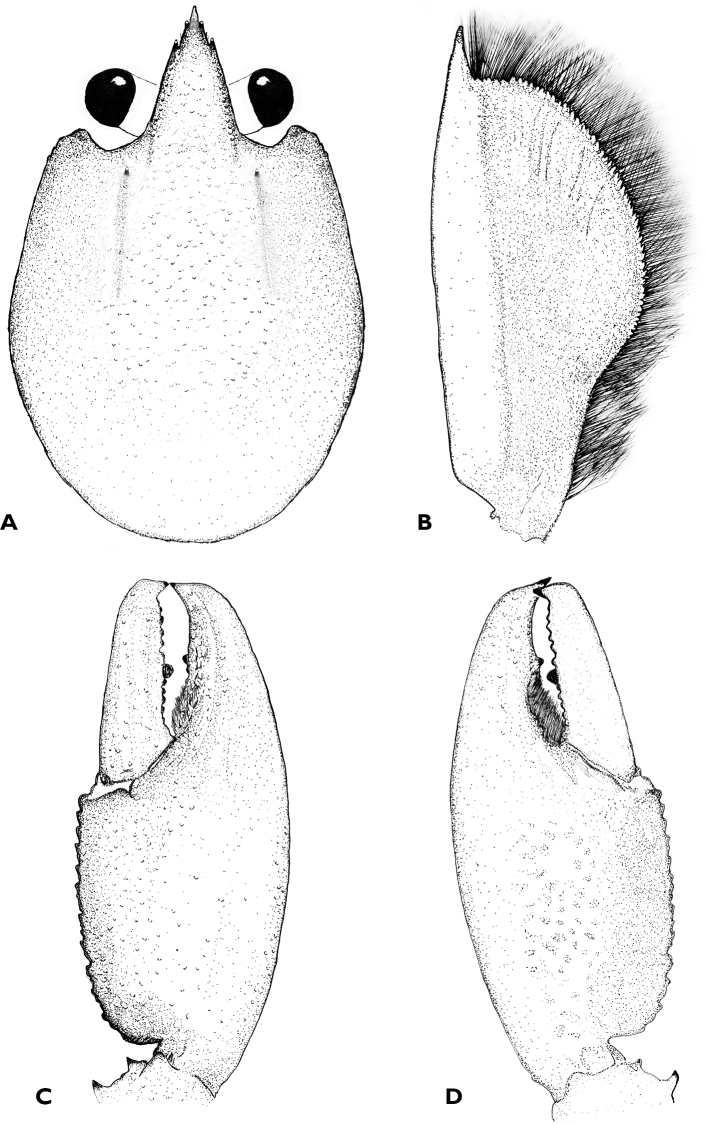
*Cherax snowden* sp. n. holotype male (MZB Cru 4291) **A** dorsal view carapace **B** scaphocerite **C** dorsal view right chela **D** ventral view left chela.

**Figure 4.**
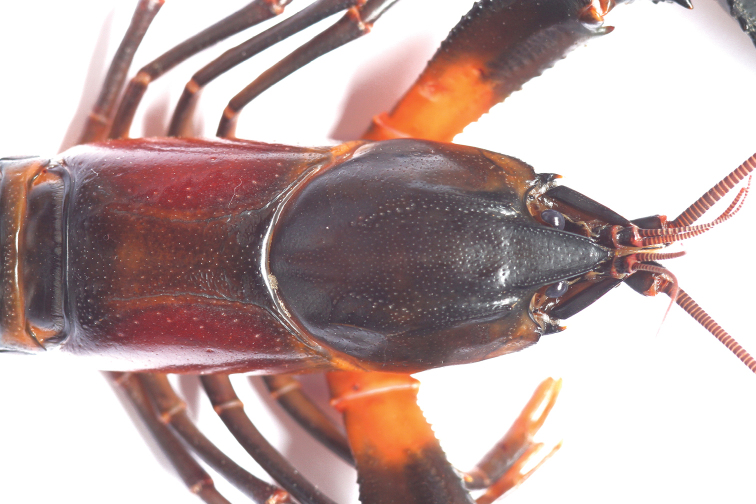
*Cherax snowden* sp. n. holotype male (MZB Cru 4291) dorsal view of cephalothorax.

**Figure 5.**
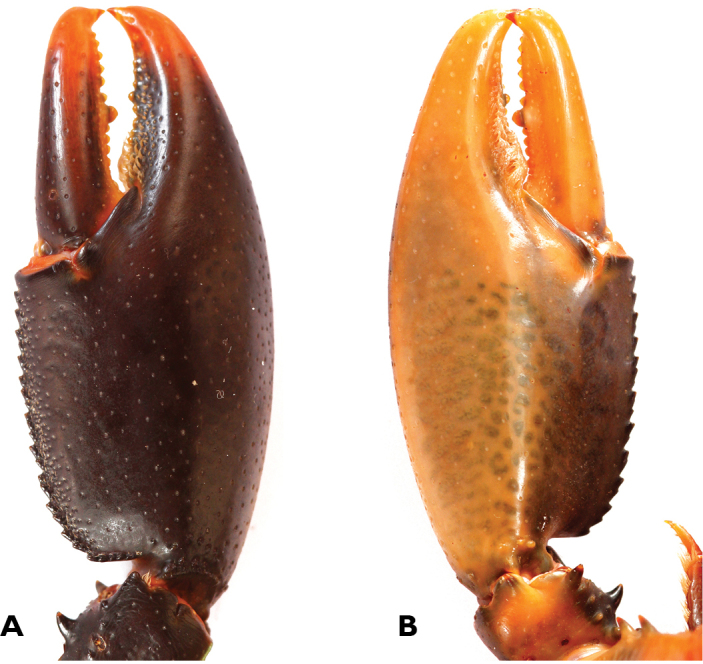
*Cherax snowden* sp. n. holotype male (MZB Cru 4291) **A** left first chela, ventral aspect **B** right first chela, dorsal aspect.

Figs 1 , 2 , 3 , 4 , 5

##### Type material.

Holotype: male (TL 96 mm) (MZB Cru 4291), Oinsok River Drainage, Sawiat District, Kepala Burung (Vogelkop) Peninsula, West Papua, Indonesia, collected by Irianto Wahid on 14 January 2015, exported through Aquazone Aquarium, Jakarta, Indonesia. Paratype: 1 male (TL 101 mm) (MZB Cru 4292), 1 allotype female (TL 77 mm) (MZB Cru 4293), same data as holotype.

##### Non-type material.

3 males (TL 69–84 mm) (MZB Cru 4294), same data as holotype.

##### Description of male holotype

(Figs 2–5). Body and eyes pigmented. Eyes not reduced.

Body subovate, slightly compressed laterally. Pleon narrower then cephalothorax (width 18 mm and 20 mm respectively). Rostrum (Fig. 3A) slender, reaching about to end of ultimate antennular peduncle and one third as long as wide (width 6 mm at base, length 9 mm). Upper surface smooth, pitted, few scattered setae present at tip of rostrum; lateral margins of rostrum almost straight in basal part, distally rather moderatly tapering towards apex. Margins slightly elevated continuing in rostral carinae on carapax. Lateral rostral margins bearing each 2 blunt spines in distal third, few short hairs present on base of rostral margins, punctaded at base. Rostral carinae extending as slight elevation posteriorly on carapace, fading shortly after beginning of postorbital ridges. Postorbital ridges well developed terminating in slightly upturned corneous spines anteriorly, fading posteriorly at two-thirds of occipital carapace length. Dorsal surface of carapace smooth, pitted, cervical and branchiocardiac grooves distinct, non-setose. Short setae present on caudal margin. Areola length 17 mm narrowest width 6 mm. Length of areola 36.95% of total length of carapace (46 mm).

Ventrolateral parts smooth with scattered pitts; anterior margin strongly produced, rounded upper margin directed inward. Dorsal surface of pleon smooth, with scattered pits; abdominal segments with short seate present on caudal margins.

Eyes rather large; cornea globular, darkly pigmented, about as long as eyestalk; eyestalk slightly narrower than cornea.

Antennulae and antennae typical for the genus. Antennae about as long as body. Antennular peduncle reaching slightly beyond acumen, antennal peduncle reaching slightly beyond apex of scaphocerite. Scaphocerite (Fig. 3B) broadest at midlength, convex in distal part becoming narrower in basal part; thickened lateral margin terminating in large corneous spine, almost reaching distal margin of ultimate segment of antennular peduncle. Right scaphocerite 8 mm long and 3,5 mm wide. Proximal margins setose. Coxicerite of antennal peduncle with spinuous tubercle anteriorly; basicerite with one lateral and one ventral spine and hooked tubercles.

Epistome broadly triangular becoming lance-shaped,with corneous spine at anterior tip, lateral surface with small tubercles; central surface smooth, excavate. Mouthparts typical for the genus.

First pereopods equal in form, chelae not gaping. subequal in size, left cheliped largest (48 mm long, 18 mm wide, 10 mm high), probably replaced. Right chelae (Fig. 3C–D) 46 mm long, 20 mm wide, 11 mm high) strongly compressed. Fingers shorter than palm (dactylus 19 mm long). Dactylus broad at base, tapering slightly towards tip, becoming about 1/2 as broad as at base. Tip with sharp, corneous, hooked tooth pointing outwards at an angle of 45°. Cutting edge of dactyl with a continuous row of rather small granular teeth and one prominent larger tooth at about middle of cutting edge. Ventral and dorsal surface of movable finger with scattered punctuation. three rows of short setae present at posterior half of the cutting edge. Fixed finger triangular, merging gradually into palm, ending in sharp, corneous, hooked tooth, standing almost perpendicular to axis of finger. Upper surface of palm practically smooth, slightly pitted, more densely pitted at margins. Five rows of short setae present in posterior part. Mesial margin of palm with a row of 23–24 tubercles. Dorsal surface of carpus (14 mm) smooth and pitted, with slight excavation in middle part. Ventral carpal surface margins slightly elevated; inner margin with set of 6-7 small granules and one acute spiniform tubercle oriented in an angle of approx 45°.

Merus (23 mm) laterally depressed in basal part; surface smooth and pitted; row of 6–7 tubercles present and a prominent spine at anterior part. Dorsolateral margin with one corneous tubercle; row of small granules on entire inner ventrolateral margin with 3 prominent spines at the anterior part. Ischium (12 mm) smooth with single granule on ventral surface.

Second pereopod reaching about to apex of scaphocerite. Finger as long as palm, of same height. Short setae present on dactyl and fixed finger, getting more dense anteriorly. Cutting edge of fixed finger and carpus with row of short setae. Carpus slightly longer than palm. Merus (15 mm) about 1.7 times longer than carpus (9 mm). Ischium (7 mm) about half as long as merus.

Third pereopod overreaching second. Fingers shorter than palm.

Fourth pereopod reaching distal margin of scaphocerite. Dactylus with corneous tip. Short setae present. Propodus more than twice as long as dactylus, about 1.5 times as long as carpus; somewhat flattened, carrying stiff setae on lower margin. Merus just slightly longer than propodus.

Fifth pereopod similar to fourth, slightly shorter.

Dorsal surface of pleon smooth in median region; pleura smooth, slightly pitted, becoming densely pitted on sixth somite and telson. Telson with posterolateral spines, dense short setae present in the posterior third. Posterior margines setose. Uropodal protopod with distal spine on mesial lobe. Exopod of uropod with two well defined spines. One distal spine on mesial lobe, with prominent median rib ending in a spine in middle of uropod. Posterior margin of proximal segment of exopod of uropod with row of small spines overlapping diaresis. Short seata present on posterior third of dorsal surface of endopod and exopod.Ventral surface of telson, endopod and exopod smooth, not pitted. Margines of exopod setose.

##### Description of paratype female

**Figure 6.**
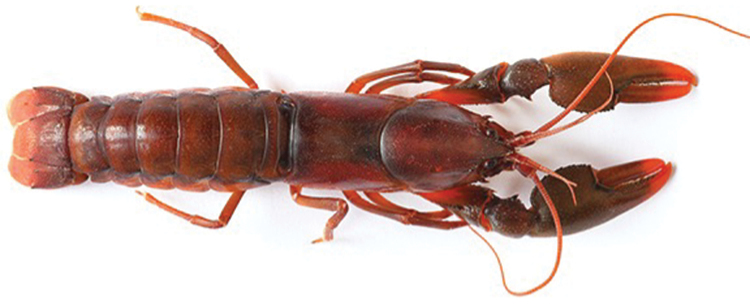
*Cherax snowden* sp. n., paratype female (MZB Cru 4293).

(Fig. 6). Chela of first pereiopods equal, about 2 times as long as broad (24 mm and 11 mm respectively). Mesial margin of palm slightly elevated, forming slender serrated ridge with row of 13-14 small granular teeth. Cutting edge of dactyl with rather small granular teeth in posterior part and one slightly larger tooth in about middle. Cutting edge of fixed finger with small granules and one slightly larger granules. Small scattered short setae visible along ventral cutting edge of chelae, more dense in posterior area. Cervical groove distinct, non setose. Cephalothorax just slightly narrower than pleon (widths 14 mm and 16 mm respectively).

**Figure 7.**
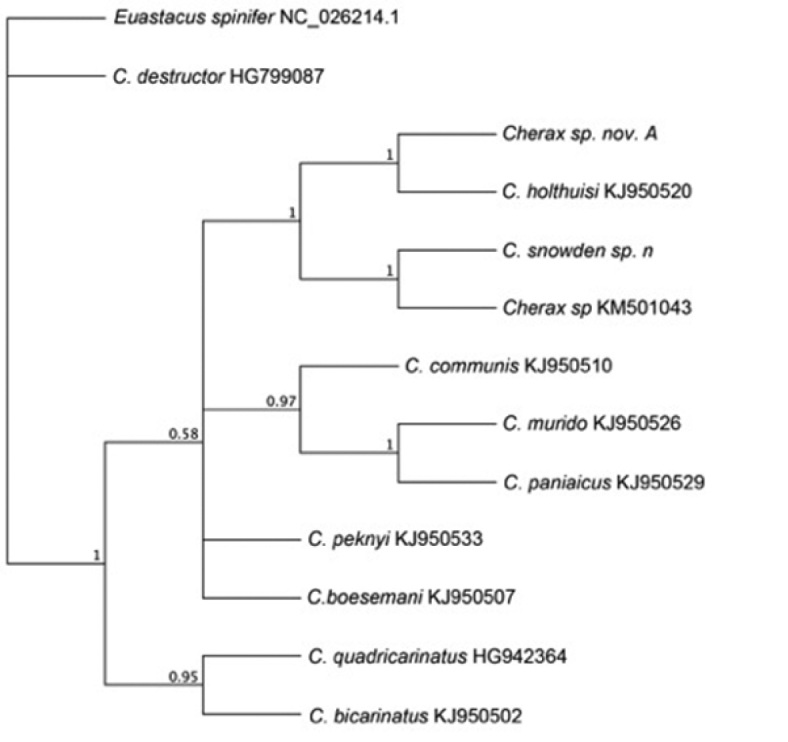
Phylogenetic consensus tree inferred from a 600 bp long fragment of COI with MrBayes. Shown are posterior probability values.

##### Size.

The males examined have a carapace length of 31–43 mm, and a total length of 69–101 mm (n = 5); the female has a carapace length of 34 mm and a total length of 77 mm (n = 1).

##### Colouration.

The living animals (Fig. 1A–C) are coloured as follows. Chelae dark green to light green or greenish gray, distal part of the lower margin cream to orange. Tips of chelae orange.

Cephalothorax dark green, light green, brown green, sometimes blueish green fading ventrally to cream, beige or orange. Pleon same colour as cephalothorax with transverse orange bands, pleura creamy to orange with a black, brown or dark green band. Walking legs from dark green to blueish gray or creamy yellow, sometimes brown yellow. Distal margin of tail-fan cream to orange.

##### Systematic position.

*Cherax snowden* sp. n. differs from *Cherax holthuisi* in the shape of the rostrum, number of rostral teeth, the shape of the chelae and coloration. While *Cherax holthuisi* has just two indentations on each side in the distal part of the rostrum and no spines present, *Cherax snowden* sp. n. has 2 rostral teeth on each side near the apex. *Cherax holthuisi* usually is orange to pale, creamy or light brown, rarerly light blue, while the new species is dark green to light green or greenish gray. Tips of the chelae in the new species are striking orange. Eyes in *Cherax holthuisi* rather small compared to the eyes of *Cherax snowden* sp. n.

The phylogenetic tree revealed that *Cherax snowden* sp. n. forms a strong supported clade with an undescribed *Cherax* sp. individual that was collected in Sorong West Papua, Indonesia (GenBank accession number: KM501043). The two sequences in this clade differ by only 9 base pair substitutions (1.5%). The low genetic divergence of the undescribed *Cherax* sp. and the close geographic sampling origin indicate that this individual is the same species as the here new described species. The *Cherax snowden* sp. n. and *Cherax* sp. clade group next to the clade which includes *Cherax* *sp. nov. A* and *Cherax holthuisi*. The species of these two neighbouring clades differ by 9.2% (*Cherax* sp. to *Cherax* *sp. nov. A*) to 9.7% (*Cherax snowden* sp. n. to *Cherax holthuisi*), respectively. The strong genetic divergence of *Cherax snowden* sp. n. to the next related described *Cherax* species indicates that *Cherax snowden* sp. n. is indeed a new species.

Holthuis (1949) in his publication on the New Guinea *Cherax* considered species should be placed into two groups. One with the rostral and median carine absent or weakly developed and refered to as the *Cherax* group following the characteristics of the type species, *Cherax preissii* (Erichson, 1846) from southwest Australia. The other group contains species that have rostral and sometimes the median carina well developed and refered to as the *Astaconephrops* group with Nobili’s (1899) *Astaconephrops albertisii* as the type. Newly described species have been placed into one or other of the two subgenera (Lukhaup and Pekny 2006, 2008; Lukhaup and Herbert 2008; Lukhaup 2015; Patoka et al. 2015). Munasinghe et al. (2004b) and Austin (1996) and Austin and Knott (1996) however identified three geographically-based lineages within *Cherax* based on molecular phylogenetic studies: a southwestern group, an eastern group and a northern group. Support for the latter group however was based on only very limited sampling (e.g. single samples of *Cherax quadricarinatus*, *Cherax rhynchotus* Riek, 1951 and *Cherax peknyi* in Munasinghe et al.’s study) (Munasinghe et al. 2004b) indicate that the division of *Cherax* into two subgenera, as conceived by Holthuis and subsequent authors dealing with New Guinea crayfish has to be reconsidered. Based on Munasinghe et al. (2004b) and Austin (1996) and Austin and Knott (1996) *Cherax snowden* sp. n. belongs to the northern species group lineage consisting of 21 species:

*Cherax albertisii*; *Cherax boesemani*; *Cherax boschmai* Holthuis, 1949; *Cherax buitendijkae* Holthuis, 1949; *Cherax communis*; *Cherax divergens* Holthuis, 1950; *Cherax gherardii* Patoka, Bláha & Kouba, 2015; *Cherax holthuisi*; *Cherax longipes* Holthuis, 1949; *Cherax lorentzi lorentzi* Roux, 1911; *Cherax lorentzi aruanus* Roux, 1911; *Cherax minor* Holthuis, 1996; *Cherax misolicus* Holthuis, 1949; *Cherax monticola* Holthuis, 1950; *Cherax murido* Holthuis, 1949; *Cherax pallidus* Holthuis, 1949; *Cherax paniaicus* Holthuis, 1949; *Cherax papuanus* Holthuis, 1949; *Cherax peknyi*; *Cherax pulcher* Lukhaup, 2015; *Cherax solus* Holthuis, 1949.

##### Etymology.

The new species is named after the american freedom fighter Edward Joseph Snowden. He is honored due to of his extraordinary achievements in defense of justice, and freedom. The name is used as a noun in apposition.

##### Ecology.

Known only from tributary creeks to the Oinsok River, Sawiat District in the central part of the Kepala Burung (Vogelkop) Peninsula. The creeks from where these crayfish have been collected are shallow (20–60 cm) with a moderate flow, the water is clear, and has a pH of approx. 6.5. In most of the parts no water plants are present. The substrate of the creek is rocky, mostly covered with silt, stones and larger rocks..To improve the knowledge of the distribution of the species more collecting trips are necessary.

It is also necessary to briefly comment on the possible threats faced by the new species. As *Cherax snowden* sp. n. is collected in large numbers for the global aquarium trade, as well as for food for the growing local population, the crayfish population will invariably be adversely impacted. According to local collectors, the populations of the species have been decreasing in the last few years. Clearly, the continued collectiing of these crayfish for the trade is not a sustainable practice, and if the popularity of the species continues, a conservation management plan will have to be developed, potentially including a captive breeding program.

## Supplementary Material

XML Treatment for Cherax snowden

## References

[B1] Ahyong ST (2014) Diversity and distribution of Australian freshwater crayfish with a check-list of the world Parastacidae and a key to the genera (Decapoda, Astacidea, Parastacoidea). Advances in Freshwater Crustacean Systematics and Biology. Crustaceana Monographs 19: 245–271.

[B2] Aljanabi SM Martinez I (1997) Universal and rapid salt-extraction of high quality genomic DNA for PCR-based techniques. Nucleic acids research 25(22): 4692–4693. doi: 10.1093/nar/25.22.4692 9358185 10.1093/nar/25.22.4692 PMC147078

[B3] Austin CM (1996) Systematics of the freshwater crayfish genus *Cherax* Erichson (Decapoda: Parastacidae) in northern and eastern Australia: electrophoretic and morphological variation. Australian Journal of Zoology 44: 259–296. doi: 10.1071/ZO9960259

[B4] Austin CM Knott B (1996) Systematics of the freshwater crayfish genus *Cherax* Erichson (Decapoda: Parastacidae) in south-western Australia: electrophoretic, morphological and habitat variation. Australian Journal of Zoology 44: 223–258. doi: 10.1071/ZO9960223

[B5] Darriba D Taboada GL Doallo R Posada D (2012) jModelTest 2: more models, new heuristics and parallel computing. Nature Methods 9(8): 772. 10.1038/nmeth.2109 PMC4594756 22847109

[B6] Erichson WF (1846) Übersicht der Arten der Gattung *Astacus*. Archiv fur Naturgeschichte 12: 86–103, 375–377.

[B7] Folmer O Black M Hoeh W Lutz R Vrijenhoek R (1994) DNA primers for amplification of mitochondrial cytochrome *c* oxidase subunit I from diverse metazoan invertebrates. Molecular Marine Biology and Biotechnology 3: 294–299. 7881515

[B8] Holthuis LB (1949) Decapoda Macrura with a revision of the New Guinea Parastacidae. Zoological results of the Dutch New Guinea Expedition 1939. No. 3 Nova Guinea (n. ser.) 5: 289–330, pls. 2–9.

[B9] Holthuis LB (1956) Native fisheries of freshwater Crustacea in Netherlands New Guinea. Contributions to New Guinea Carcinology. I. Nova Guinea (n. ser.) 7(2): 123–137, figs 1–3, pls. 1–8.

[B10] Holthuis LB (1958) Freshwater Crayfish in Netherlands New Guinea Mountains. South Pacific Commission Quarterly Bulletin 8(2): 36–39, 7 figs.

[B11] Holthuis LB (1982) Freshwater Crustacea Decapoda of New Guinea. In: Gressitt JL (Ed.) Biogeography and ecology of New Guinea, vol. 2 Monographiae Biologicae 42: 603–619, figs 1–5. doi: 10.1007/978-94-009-8632-9_28

[B12] Holthuis LB (1986) The freshwater crayfish of New Guinea. Freshwater Crayfish 6: 48–58, figs 1–8.

[B13] Holthuis LB (1996) Cherax (Astaconephrops) minor new species, a parastacid from the mountains of Irian Jaya (W. New Guinea) Indonesia (Crustacea: Decapoda: Parastacidae). Zoologische Mededelingen Leiden 70(24): 361–366, figs 1–2.

[B14] Huxley TH (1879) The Crayfish: an Introduction to the Study of Zoology. C. Kegan Paul & Co., London.

[B15] Lukhaup C (2015) Cherax (Astaconephrops) pulcher, a new species of freshwater crayfish (Crustacea, Decapoda, Parastacidae) from the Kepala Burung (Vogelkop) Peninsula, Irian Jaya (West Papua), Indonesia. ZooKeys 502: 1–10. doi: 10.3897/zookeys.502.9800 26019660 10.3897/zookeys.502.9800 PMC4443586

[B16] Lukhaup C Herbert B (2008) *Cherax peknyi* sp. nov., a new species of crayfish (Crustacea: Decapoda: Parastacidae) from the Fly River Drainage, Western Province, Papua New Guinea. Memoirs of the Queensland Museum 52(2): 213–219.

[B17] Lukhaup C Pekny R (2006) *Cherax holthuisi*, a new species of crayfish (Crustacea: Decapoda: Parastacidae) from the centre of the Vogelkop Peninsula in Irian Jaya (West New Guinea), Indonesia. Zoologische Mededelingen Leiden 80–1(7): 101–107, figs 1–4.

[B18] Lukhaup C Pekny R (2008a) Cherax (Astaconephrops) boesemani, a new species of crayfish (Crustacea: Decapoda: Parastacidae) from the centre of the Vogelkop Peninsula in Irian Jaya (West New Guinea), Indonesia. Zoologische Mededelingen Leiden 82(33): 331–340, figs 1–8.

[B19] Lukhaup C Pekny R (2008b) Süßwasserkrebse aus aller Welt Gebundene Ausgabe. Dähne Verlag, Ettlingen.

[B20] Lukhaup C Pekny R (2014) Wirbellose - Garnelen, Krebse, Krabben & Schnecken im Süßwasseraquarium. Gebundene Ausgabe. Dähne Verlag, Ettlingen, 300 pp.

[B21] Munasinghe DHN Burridge CP Austin CM (2004a) The systematics of freshwater crayfish of the genus *Cherax* Erichson (Decapoda: Parastacidae) in eastern Australia re-examined using nucleotide sequences from 12S rRNA and 16S rRNA genes. Invertebrate Systematics 18: 215–225. doi: 10.1071/IS03012

[B22] Munasinghe DHN Burridge CP Austin CM (2004b) Molecular phylogeny and zoogeography of the freshwater crayfish genus *Cherax* Erichson (Decapoda: Parastacidae) in Australia. Biological Journal of the Linnean Society 81: 553–563. doi: 10.1111/j.1095-8312.2003.00299.x

[B23] Patoka J Blaha M Kouba A (2015) Cherax (Astaconephrops) gherardii, a new crayfish (Decapoda: Parastacidae) from West Papua, Indonesia. Zootaxa 3964(5): 526–536. doi: 10.11646/zootaxa.3964.5.2 26249463 10.11646/zootaxa.3964.5.2

[B24] Ronquist F Huelsenbeck JP (2003) MrBayes version 3.0: Bayesian phylogenetic inference under mixed models. Bioinformatics 19(12): 1572–1574. doi: 10.1093/bioinformatics/btg180 12912839 10.1093/bioinformatics/btg180

